# Tibial Baseplate Migration Is Not Associated with Change in Patient-Reported Outcome Measures and Clinical Scores After TKA: A Secondary Analysis of 5 Radiostereometric Analysis Studies with 10-Year Follow-up

**DOI:** 10.2106/JBJS.23.00957

**Published:** 2024-06-28

**Authors:** Thies J.N. van der Lelij, Bart L. Kaptein, Roula Tsonaka, Rob G.H.H. Nelissen, Sören Toksvig-Larsen, Perla J. Marang-van de Mheen

**Affiliations:** 1 Department of Orthopaedics, Leiden University Medical Center, Leiden, The Netherlands; 2 Department of Biomedical Data Sciences, Medical Statistics, Leiden University Medical Center, Leiden, The Netherlands; 3 Department of Orthopaedics, Hässleholm Hospital, Hässleholm, Sweden; 4 Department of Clinical Sciences, Lund University, Lund, Sweden; 5 Safety and Security Science, Centre for Safety in Healthcare, Delft University of Technology, Delft, The Netherlands

## Abstract

**Background:**

Radiostereometric analysis (RSA) provides highly accurate data about the migration of a total knee arthroplasty (TKA) component. However, patient-reported outcome measures (PROMs) reflect the patients’ perspective of their functional status, pain, and overall health after TKA. The aim of this study was to evaluate the association between tibial implant migration and change in postoperative PROMs and clinical scores, using data pooled from long-term follow-up RSA studies.

**Methods:**

Individual implant migration data were collected from 5 randomized RSA studies, including a total of 300 patients with 6 distinct TKA implant designs (all Stryker). Tibial implant migration (maximum total point motion [MTPM]) was evaluated with RSA at 3 months, 1 year, and 2, 5, 7, and 10 years postoperatively. The Knee Society Score (KSS)-Knee and KSS-Function and Knee Injury and Osteoarthritis Outcome Score (KOOS) subscales were collected in all studies at the same follow-up times. Linear mixed-effects models, with adjustment for TKA implant design and patient characteristics, were used to analyze the data. The 3-month follow-up visit was used as the baseline to assess the association between implant migration and PROMs across the 10-year follow-up.

**Results:**

No association between tibial implant migration and change in KSS-Knee (p = 0.384), KSS-Function (p = 0.737), KOOS-Symptoms (p = 0.398), KOOS-Pain (p = 0.699), KOOS-Activities of Daily Living (p = 0.205), KOOS-Sport and Recreation (p = 0.702), or KOOS-Quality of Life (p = 0.368) was found across the entire follow-up. Similar results were found when using the 2-year follow-up as the baseline, after which both cemented and uncemented implants are expected to have stabilized.

**Conclusions:**

Tibial baseplate migration was not associated with postoperative worsening in PROMs or clinical scores in patients who underwent TKA. These findings suggest that implant migration, as measured with RSA, measures a different parameter (i.e., implant-bone fixation) than PROMs (i.e., patient perception) and clinical scores. Therefore, to assess the performance and safety of TKA implant designs, RSA and PROMs cannot be used interchangeably during the postoperative follow-up of patients and evaluation of the fixation of knee implants.

**Level of Evidence:**

Prognostic Level III. See Instructions for Authors for a complete description of levels of evidence.

Patient-reported outcome measures (PROMs) are increasingly employed in orthopaedics, reflecting the change of focus from volume-based to value-based health-care delivery by evaluating what matters and what is expected by patients after arthroplasty^1-4^. Given the increasing health-care costs of and the excellent performance after most total knee arthroplasties (TKAs), PROMs have been suggested as a feasible alternative to the traditional regular outpatient clinic follow-up after TKA^5,6^.

Radiostereometric analysis (RSA) is a highly accurate and objective technique to detect minimal implant migration (0.1 to 0.2 mm) during early follow-up, which is associated with implant (e.g., TKA implant) revision risk^7,8^. If TKA implant migration (i.e., implant fixation in the bone) is associated with a decrease in PROMs and/or clinical knee scores, this would suggest that these scores can be used interchangeably for the follow-up of patients who underwent TKA, thereby reducing costs (e.g., no clinical visits) while maintaining quality and safety for the patients who underwent TKA. To our knowledge, no studies have investigated the association of tibial baseplate migration in patients who underwent TKA and PROMs and clinical scores. Recently, Steiner et al. found that hip stem migration did not significantly influence PROMs at 2 years postoperatively in patients who underwent total hip arthroplasty (THA)^9^.

The aim of the present study was to assess whether TKA tibial component migration, as measured with RSA, is associated with changes in postoperative PROMs and clinical scores in patients who undergo TKA. We hypothesized that tibial implant migration is not associated with postoperative improvement in PROMs or clinical scores, as they measure different constructs.

## Materials and Methods

### Study Design

Pooling individual tibial baseplate migration data from multiple, long-term RSA studies increases the statistical power to detect possible associations^10^. Long-term follow-up data were collected from 5 individual RSA studies, all conducted at a single center (Hässleholm Hospital) with inclusion periods between 2006 and 2010 (Table I). Patient selection, baseline characteristics, and surgical procedures of the studies have been described in previous short-term and mid-term reports^11-17^. In short, each study was a randomized controlled trial (RCT) using RSA to assess differences in migration between 2 TKA implant designs. The studies included 300 patients in total and 6 distinct TKA implant designs. The Triathlon cruciate-retaining (CR) cemented implant was included in 4 studies, and the Triathlon CR uncemented peri-apatite (PA)-coated implant was included in 2 studies. The other TKA designs were included in 1 study each: the Duracon CR cemented, Triathlon posterior-stabilized (PS) cemented, Triathlon CR uncemented porous-coated, and Triathlon short-stem (i.e., short-keeled) CR cemented implants (all Stryker).

**TABLE I:** Study Characteristics

	Implant Designs	Inclusion Period	No. of Patients	ClinicalTrials.gov Registration
Study 1^11,12^	Triathlon CR cemented, Duracon CR cemented	2006	60	NCT00436982
Study 2^13^	Triathlon CR cemented, Triathlon PS cemented	2007	60	NCT02522728
Study 3^14,15^	Triathlon CR uncemented PA-coated, Triathlon CR uncemented porous-coated	2007 to 2008	60	NCT03198533
Study 4^16^	Triathlon CR cemented, Triathlon short-stem CR cemented	2008 to 2010	60	NCT02525614
Study 5^17^	Triathlon CR cemented, Triathlon CR uncemented PA-coated	2009 to 2010	60	NCT02525601

### RSA

In all studies, RSA radiographs were made on the first day after the surgical procedure when weight-bearing was achieved. Subsequent examinations were performed at 3 months, 1 year, and 2, 5, 7, and 10 years postoperatively. RSA radiographs were made with the patient in a supine position with the knee in a calibration cage (Cage 10; RSA Biomedical). Migration was calculated using marker-based analysis, with 8 tantalum beads with a diameter of 0.8 mm (RSA Biomedical) inserted into the tibial bone and 5 beads inserted into the polyethylene insert. The same experienced RSA analyst performed the migration calculations in all studies using all available markers at each follow-up that could be matched to the baseline RSA image. The postoperative RSA examination served as the reference for migration calculations in all studies. Analyses were performed with UmRSA software (version 6.0; RSA Biomedical) in concordance with the International Organization for Standardization (ISO) standard and RSA guidelines^18,19^. Maximum total point motion (MTPM), which is the length of the translation vector of the marker in a rigid body with the greatest migration, was used as the primary outcome measure for implant migration.

### PROMs and Clinical Scores

The Knee Society Score (KSS) and Knee Injury and Osteoarthritis Outcome Score (KOOS) were obtained preoperatively and at 3 months, 1 year, and 2, 5, 7, and 10 years postoperatively in each study^20,21^. The KSS can be divided into the KSS-Knee and the KSS-Function. The KOOS has 5 separately scored subscales: Symptoms, Pain, Activities of Daily Living, Sport and Recreation (SR), and Quality of Life (QoL). All scores can range from 0 to 100, with higher scores indicating better outcomes. Only the KSS-Knee requires clinical assessment of the knee, including the assessment of the range of motion and stability, and was therefore considered to be a clinical score. All patient-reported outcome scores were obtained from validated questionnaires.

### Ethics and Registration

All studies were approved by the local ethics committee^11-17^ and registered at ClinicalTrials.gov (Table I), and all patients gave their informed consent. A protocol to pool the data from the studies was presented to the medical ethics committee of Leiden University Medical Center, which waived the need for approval under Dutch law (P.15.198).

### Statistical Analysis

A linear mixed-effects model (LMM) was used to assess the MTPM of each specific TKA implant design over the 10-year follow-up period, as this model takes the correlation of measurements performed on the same patient into account and deals effectively with missing values during follow-up; for patients who withdrew from the study (e.g., due to revision), all measurements until withdrawal were included^22,23^. MTPM was log-transformed, computed as log_10_(MTPM + 1), to obtain a normally distributed variable. The presented values have been back-transformed to the original scale. To assess the PROMs for the different TKA implant groups at the specific follow-up times, a comparable generalized estimating equation (GEE) approach was used, as a normal distribution could not be obtained through transformation.

To assess the association of tibial baseplate migration (MTPM) with PROMs and clinical scores, separate LMMs were used for the different subscores (KSS-Knee, KSS-Function, KOOS-Symptoms, KOOS-Pain, KOOS-ADL, KOOS-SR, or KOOS-QoL). The 3-month follow-up visit was used as the baseline, as this was the first follow-up time in which RSA examinations and PROMs were collected at the same time. The changes in PROMs and clinical scores at each follow-up visit were calculated, as well as the tibial implant migration (MTPM) relative to the 3-month follow-up. The models included a PROM variable (change in score relative to the 3-month follow-up score), a time variable (3 months, 1 year, and 2, 5, 7, and 10 years), and an interaction term between time and the PROM to reflect that PROM improvement and thereby its association with migration might change over time. TKA implant design was included as a fixed factor, to account for the possible influence of implant design and fixation method. Baseline patient characteristics (age, sex, American Society of Anesthesiologists [ASA] score, and body mass index [BMI]) were added to the model as fixed factors as well. For the random-effects structure, a random-intercept term was used and the remaining variability was modeled with an autoregressive order-1 covariance structure. Beyond 2 years, after the initial settling phase, both cemented and uncemented implants should not show any progression in migration. Continuous implant migration beyond 2 years indicates insufficient fixation in the bone, and these implants are considered at risk for future aseptic loosening. Therefore, we additionally assessed the association between implant migration and PROMs and clinical scores after 2 years postoperatively. Means were reported with 95% confidence intervals (CIs), and significance was set at p < 0.05. Analyses were performed using SPSS (version 26.0; IBM) and R software (version 4.1.0; The R Foundation).

## Results

**Fig. 1:**
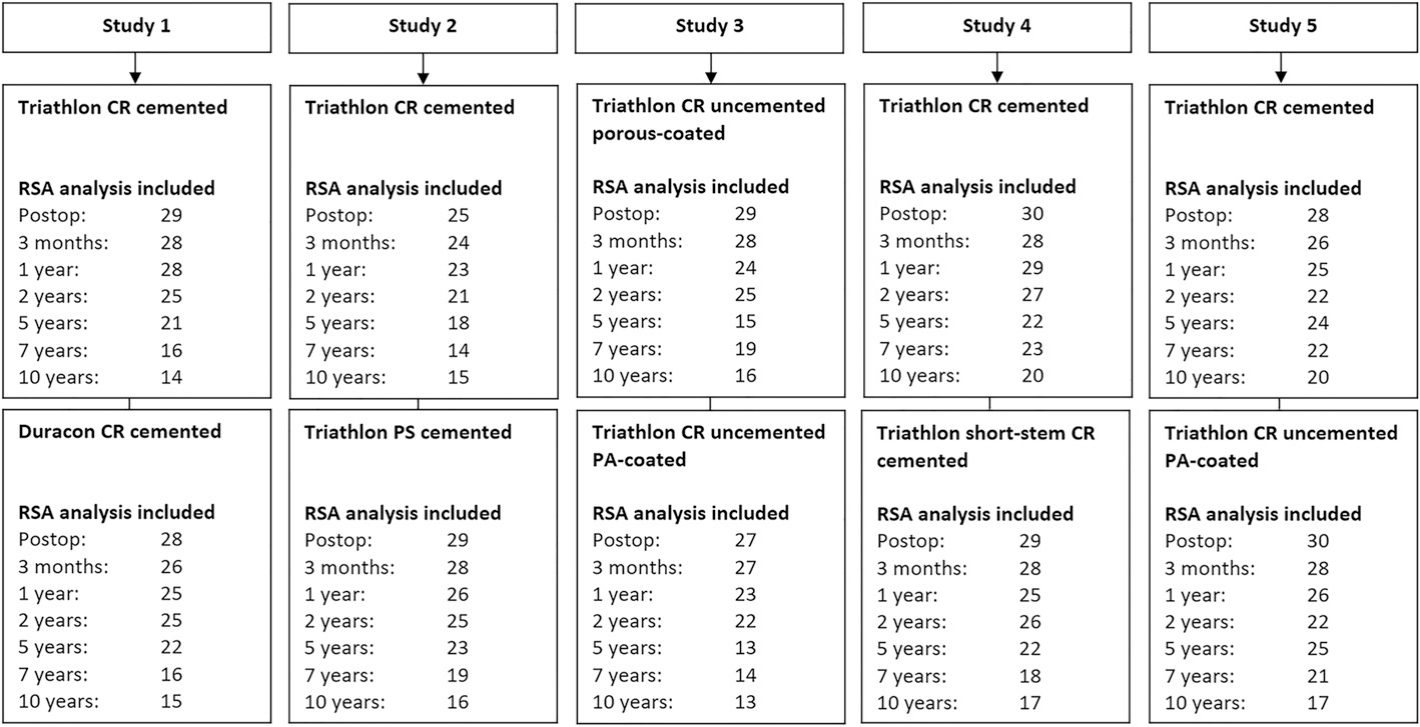
Number of RSA examinations included in the present study at each follow-up time for each individual RCT.

A total of 300 patients were initially included in all studies. During the 10-year follow-up period, 7 implants were revised because of infection (n = 2), component loosening (n = 2), instability (n = 1), or insert wear (n = 2). The number of RSA examinations included in our analysis is presented in Figure 1. At the 10-year follow-up, RSA migration data were available for 163 patients (Fig. 1). The complete CONSORT (Consolidated Standards of Reporting Trials) flow diagrams of all individual studies are provided in Appendix Figure A-1. The most common reasons for not including RSA measurements were inadequate quality of radiographs (e.g., not adhering to the RSA guidelines and the ISO standard) or missing RSA radiographs of patients who remained included in the study (e.g., a missed follow-up time)^18,19^.

### RSA Migration Measurements

**Fig. 2:**
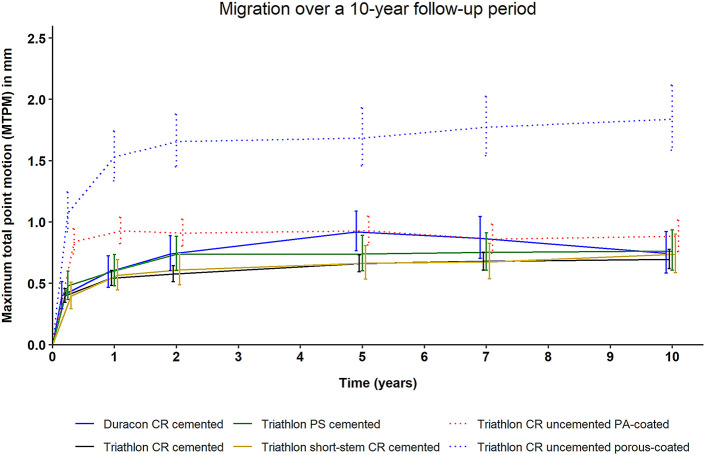
The mean MTPM, derived from the LMM analysis, during the 10-year follow-up. The error bars indicate the 95% CIs. The individual lines represent distinct TKA implant designs.

The different TKA implant designs showed distinct long-term migration patterns, with the Triathlon CR uncemented porous-coated TKA implant showing the highest absolute migration (i.e., MTPM) throughout the follow-up period (Fig. 2). At the 10-year follow-up, the mean migration of this TKA implant was 1.84 mm (95% CI, 1.59 to 2.12 mm) compared with 0.74 mm (95% CI, 0.58 to 0.92 mm) for the Duracon CR cemented TKA implant, 0.70 mm (95% CI, 0.62 to 0.78 mm) for the Triathlon CR cemented TKA implant, 0.76 mm (95% CI, 0.61 to 0.94 mm) for the Triathlon PS cemented TKA implant, 0.74 mm (95% CI, 0.59 to 0.90 mm) for the Triathlon CR short-stem cemented TKA implant, and 0.88 mm (95% CI, 0.76 to 1.02 mm) for the Triathlon CR uncemented PA-coated TKA implant. There was no difference in migration pattern (i.e., initial implant migration and later stabilization) of the Triathlon CR cemented TKA implant within the 4 studies in which this implant was included (p = 0.98). Also, the Triathlon CR uncemented PA-coated TKA implant showed a comparable migration pattern during the 10-year follow-up in the 2 studies evaluating this design (p = 0.99) (see Appendix Fig. A-2).

### Association of Tibial Implant Migration with PROMs and Clinical Scores

The LMMs showed no significant association between KSS-Knee (p = 0.384), KSS-Function (p = 0.737), KOOS-Symptoms (p = 0.398), KOOS-Pain (p = 0.699), KOOS-ADL (p = 0.205), KOOS-SR (p = 0.702), or KOOS-QoL (p = 0.368) and TKA tibial component migration during the 10-year follow-up when 3 months postoperatively was used as the baseline measure. Analyzing tibial baseplate migration beyond 2 years postoperatively (i.e., when implants are expected to show no progression in migration), no association with KSS-Knee (p = 0.063), KSS-Function (p = 0.169), KOOS-Symptoms (p = 0.174), KOOS-Pain (p = 0.476), KOOS-ADL (p = 0.424), and KOOS-SR (p = 0.764) could be found. Only a significant association between MTPM and change in KOOS-QoL between 2 and 10 years of follow-up was found (p = 0.045). The mean PROMs and clinical scores for each TKA implant design at the specific follow-up times are presented in Appendix Table A-I.

## Discussion

No association between TKA tibial component migration and postoperative change in PROMs or clinical scores of patients during the 10-year follow-up after TKA was found in the present study, meaning that worsening or less improvement of postoperative PROMs and clinical scores over time does not indicate greater TKA implant migration. Tibial component migration likely measures something different (i.e., the fixation of the implant in the bone) than PROMs (i.e., patient perception) or clinical scores. Thus, when evaluating implant performance, migration cannot be used interchangeably with PROMs or clinical scores. Furthermore, at an individual patient level, PROMs will not provide the ability to detect implant loosening at an early stage, which would be necessary for newly introduced implants to have a guarantee of clinical benefit (i.e., pain relief, function, and bone fixation) and patient safety.

For the primary analysis, the 3-month follow-up visit was used as the baseline, as this was the first follow-up time at which RSA examinations and PROMs were collected at the same follow-up time point. Although the greatest improvement in PROMs and clinical scores relative to the preoperative period generally occurs within the first few months after TKA, this was not of interest for the present study, as our goal was to assess whether implant migration (as measured with RSA) and PROMs could be used interchangeably during a 10-year, long-term follow-up period of patients who underwent TKA. No association between MTPM and any of the PROMs or clinical scores was found in our primary analyses. Using the 2-year follow-up as the baseline, there was no association between MTPM and PROMs or clinical scores, except for a marginally significant association between MTPM and KOOS-QoL (p = 0.045). However, the coefficients of the change in KOOS-QoL and of the interaction term of time with the change in KOOS-QoL, as derived from the specific LMM, were both very small and were well outside the range of clinically relevant changes in MTPM that could predict aseptic loosening. Also, even though the Triathlon CR uncemented porous-coated TKA implant group showed the greatest mean migration (Fig. 2), the mean KOOS-QoL at all follow-up times was comparable or even slightly higher compared with the other TKA implant groups (see Appendix Table A-I).

The societal pressure to control health-care costs has prompted increased emphasis on PROMs as a measure of the outcome of treatment. Although patients’ opinions are important, associations with more objective measures of treatment outcome are complex. PROMs have been used as an easy method to control health-care costs in value-based health-care initiatives, where value is measured as dollars relative to quality of care^24^. Value-added care is more complex than simply the relation between money and 1 outcome measure, even more so because the outcome measure is a subjective measure such as PROMs, which are complex entities^1,25^. There is a lack of consensus on specific score differences for the various PROMs that are clinically important or important to patients^26-28^. Nevertheless, defining a “successful” TKA for a patient is important, although a single validated, reliable, and responsive questionnaire addressing the priorities of patients who underwent TKA has been elusive^29^. That elusiveness is related to the multidimensional aspects of outcomes, which are related to the implant-bone fixation (i.e., to the more technical aspects of implant surgery) as well as to whether the patients still have symptoms and whether the surgical procedure met their preoperative expectations^1^. Moreover, various patient factors, including age, sex, BMI, and psychological factors, have been suggested to influence the improvement in patient-reported outcomes^30-33^. Improvement in PROMs after TKA can be related to aspects other than the prosthesis itself, such as the patient’s social context and other patient factors. However, the performance of the implant, such as fixation or loosening, can be measured objectively by RSA. Furthermore, cutoff values for MTPM show implants that are at risk for loosening (and therefore warrant close monitoring).

Clinical RSA studies only need a small number of patients to achieve adequate power, because of the high accuracy of the technique^19^. In addition to the primary outcome of implant migration, clinical RSA studies frequently collect multiple PROMs and clinical scores at each follow-up time as secondary outcomes. Collecting these questionnaire responses and clinical scores is a time-consuming and expensive process. However, individual RSA studies are not powered to detect differences in PROMs or clinical scores between TKA implant groups, raising questions regarding the purpose of collecting these scores in the clinical RSA trials. Also, the accuracy of RSA has been described as 0.1 to 0.2 mm, raising the question of whether such small micromotion could translate to clinical symptoms that would indicate an increased risk of implant failure due to aseptic loosening^34^. Several RSA migration thresholds, based on either mean migration in TKA implant groups or migration of individual implants, have been described in the literature as being associated with increased risk of revision due to loosening^7,34-36^. For example, TKA implants with a mean migration between 0.5 and 1.6 mm are considered to be at risk of having revision rates of >5% at 10 years^7^. As for individual implants, MTPM of ≥0.3 mm between 2 and 5 years is often used to classify individual implants as continuously migrating and at risk for revision due to loosening^34^.

The present study has several strengths. First, by pooling individual patient data (RSA migration data, differences in PROMs, and clinical scores) from multiple studies, the sample size and statistical power were increased. Second, 10-year follow-up RSA migration studies are scarce, as most studies remain limited to 2-year follow-up. Third, detection of implant loosening on standard radiographs by clinicians differs from measurement of implant migration as measured with RSA. Whereas loosening as identified by clinicians is subjective and categorizes implants as either loose or stable, RSA provides highly accurate and objective implant migration measurements on a continuous scale and can detect excessive migration before patients experience clinical symptoms. Finally, all included studies used the same marker-based RSA method and every examination was analyzed by the same experienced RSA analysist, using the same software and marker-selection method, increasing comparability between studies.

This study also had limitations. First, a limited number of TKA implant designs from a single manufacturer were included, which may have limited the generalizability of the results to other designs, although the concept of implant fixation in the bone is a generic principle of all well-performing orthopaedic implants. Second, loss to follow-up was present in all studies. This may have biased the association between tibial implant migration and changes in PROMs if patient withdrawal was related to worsening PROMs or migration. However, only a few patients underwent a revision surgical procedure and the main reason for fewer RSA measurements at later follow-up points involved the quality of RSA radiographs. Furthermore, all data from patients withdrawn from the study were still included in the analysis until their last available follow-up, which will have minimized any bias that might occur.

In conclusion, the lack of association between implant migration and changes in postoperative PROMs or clinical scores suggests that implant migration measures something different (i.e., the implant-bone fixation) than PROMs (i.e., patient function) and clinical scores. This suggests that both are needed for a comprehensive evaluation of TKA implant performance and they cannot be used interchangeably in the follow-up of patients who underwent TKA. Future studies should address whether our findings can be generalized to other arthroplasty implant designs.

## Appendix

Supporting material provided by the authors is posted with the online version of this article as a data supplement at jbjs.org (http://links.lww.com/JBJS/I87).
